# Worldwide trends in mortality related to Parkinson's disease in the period of 1994–2019: Analysis of vital registration data from the WHO Mortality Database

**DOI:** 10.3389/fneur.2022.956440

**Published:** 2022-10-04

**Authors:** Ioannis C. Lampropoulos, Foteini Malli, Olga Sinani, Konstantinos I. Gourgoulianis, Georgia Xiromerisiou

**Affiliations:** ^1^Respiratory Medicine Department, Faculty of Medicine, University of Thessaly, Larissa, Greece; ^2^Respiratory Disorders Laboratory, Faculty of Nursing, University of Thessaly, Larissa, Greece; ^3^Department of Neurology, School of Medicine, University of Thessaly, Larissa, Greece

**Keywords:** Parkinson's disease, mortality, trends, epidemiology, neurodegenerative disorders

## Abstract

**Background:**

Mortality due to Parkinson's disease (PD) and its long-term trends worldwide in recent decades remain unknown. No previous studies have simultaneously studied age- and sex-specific mortality trends at a population level worldwide. Insights gained from this study can help identify high-risk populations and inform healthcare service requirements for managing Parkinson's disease globally.

**Objectives:**

The aim of the study was to examine trends in mortality from Parkinson's disease by age-group and sex across countries all over the world. In this study, we used worldwide registry data to examine the temporal trends in PD mortality across most counties of the world from 1994 to 2019 using joinpoint regression.

**Results:**

In data from vital registration systems, huge variations in the patterns of deaths due to Parkinson's disease were observed both over time and between countries. Between 1994 and 2019, there was a significant increase in mortality rates globally in both men and women. In more detail, the mortality rate (per 100,000) in 1994 was 1.76 and reached 5.67 in 2019. Greater increases in mortality were seen in men than in women; and in older than in younger people.

**Conclusions:**

There has been a striking rising trend in Parkinson's disease mortality globally. Persistent age and sex disparities are found in Parkinson's disease mortality trends. Our findings may have important implications for future research, healthcare planning, and provision.

## Introduction

Parkinson's disease (PD) is the second most common neurodegenerative disorder after Alzheimer's disease, and is characterized by several motor and nonmotor symptoms that accumulate over time (1). Nowadays, PD has become one of the main causes of disability worldwide, which places a substantial burden on individual and social levels (1, 2).

Several studies report data on the epidemiology of PD. It is generally accepted that the prevalence of the disease ranges from 1 to 2 per 1,000 individuals in unselected populations and that the disease affects 1% of the population older than 60 years (3). PD is rare before the age of 50 years and reaches a prevalence of 4% in the highest age-groups (4).

The incidence of the disease varies considerably in different reports. This is probably due to methodological differences, in particular differences in case ascertainment and use of diagnostic criteria. The annual incidence per 100,000 inhabitants ranges from < 10 to more than 20. Incidence studies may be affected by under-diagnosing of PD, especially among the most elderly (5).

Many studies have depicted the rising incident trends of PD, which might be related to population growth, aging, and many environmental causes as well as to improved diagnostic methods nowadays (3).

One important aspect of PD prognosis is mortality, which has been reviewed nonsystematically and systematically in several studies (6, 7). These studies showed generally increased but markedly heterogeneous mortality ratios with no formal analysis of the cause of the heterogeneity. Recently, a meta-analysis of mortality in PD has been published, but this included a restricted number of studies and excluded retrospective studies and studies reporting unadjusted risk (8).

However, to the best of our knowledge, the mortality from PD and its long-term trends worldwide in recent decades remain unknown. Updating vital statistics about PD mortality is critical to inform future research, in priority setting, in financing of healthcare, and in setting policy. In this study, we used worldwide registry data to examine the temporal trends in PD mortality across most counties of the world from 1994 to 2019.

## Materials and methods

### Data source

World Health Organization (WHO) Global Health Estimates provide the latest available data on causes of death and disability globally, by region and country, age, sex, and income group.

These estimates are produced using data from multiple sources, including national vital registration data, latest estimates from WHO technical programs, United Nations partners and inter-agency groups, the Global Burden of Disease, and other scientific studies. Before publishing, the GHE are reviewed by WHO member states *via* consultation with national focal points and WHO country and regional offices.

We performed an analysis of vital registration data from the WHO Mortality Database (1994–2019). The WHO Mortality Database incorporates member state-level data grouped by age and sex for the primary causes of death. These data are based on medically certified reports submitted annually to the WHO by member states, which require legal certification of death as standard International Classification of Disease (ICD) codes and therefore rely on national vital registration systems. The information transmitted by each member state is validated according to standard procedures, including plausibility check, agreement with certain distributions and previous years, and validation against the ICD codes. WHO Mortality Database data have been used previously for analysis of time trends in cerebrovascular, cardiovascular, and respiratory causes of death and in the Global Burden of Disease Study. In this analysis, we focused on Parkinson's disease-related mortality and its contribution to total mortality calculated in the WHO European Region for the most recent period available in the WHO Mortality Database (2013–19) and on time trends across age-groups, sexes, and subregions from 2000 to 2019.

### Definition of Parkinson's disease-related death

Deaths were considered related to Parkinson's disease if either the ICD 10th Revision G20 code specific for Parkinson's disease was listed as the primary cause of death, defined as “the disease or event that started the chain of events that led to death.”

### Data extraction

We extracted the number of Parkinson's disease-related deaths, total deaths, and population size from the WHO Mortality Database (latest update December, 2019) for the period from 1994 (or the first available year) to 2019 (or the last available year if it preceded 2019) (9). We chose this particular period based on the following considerations: homogeneous and sensitive diagnostic criteria, standardized treatment regimens, and evidence-based management guidelines only became widely available after 1990.

Information on secondary causes of death, ethnic origin, and socioeconomic status is not available in the WHO Mortality Database. We followed the Guidelines for Accurate and Transparent Health Estimates Reporting standards.

This study did not need ethics or institutional review board approval.

### Statistical analysis

We calculated crude annual average Parkinson's disease mortality rates for 2000–2019 and for individual years between 1994 and 2019 by dividing the number of Parkinson's disease-related deaths by the total corresponding population. We calculated annual mortality rates per 100,000 population and the corresponding 95% CI (exact Poisson) (10).

We also calculated mortality rates and average mortality rates according to a classification of countries based on the human Development Index (HDI). The Human Development Index (HDI) is a statistic composite index of life expectancy, education (mean years of schooling completed and expected years of schooling upon entering the education system), and per capita income indicators, which are used to rank countries into four tiers of human development: very high, high, medium, and low human development.

Because age-groups were reported differently across countries, we created homogeneous 5-year age-groups up to 85 years. We considered zero counts as true zeroes in calculations. For the analysis of Parkinson's disease-related mortality (1994–2019, annual average), missing population data for single years were calculated from the slope obtained for the other two available years. To obtain the age-specific Parkinson's disease-related mortality for entire WHO continents or regions, we calculated unweighted and weighted (random effects model) pooled mortality rates. We used smoothed lines generated by locally estimated scatterplot smoothing to depict the proportion of deaths attributed to pulmonary embolism (proportionate mortality), reported as the number of Parkinson's disease-related deaths per 1,000 deaths.

To analyze time trends in Parkinson's disease-related mortality from 1994 to 2019, we did not apply any imputation technique for each country with missing data. Each country contributed to the analysis only for the years with complete data; we performed sensitivity analyses accounting for different thresholds of missing data and excluding member states accordingly. We reported pooled mortality rates, weighted by the population size of each member state, and corresponding 95% CIs using a locally estimated scatterplot smoothing procedure. We used joinpoint regression (JoinPoint version 4.6.0.0) to identify changes in trends and time points with significant inflections on the basis of a series of hypothesis tests (8, 10). We carried out sensitivity analyses with different assumptions concerning error variance and calculated the average annual percentage change with corresponding 95% CIs. We used a standard significance threshold of 5% for *p*-values, which depicts the probability of observing more extreme data under the null hypothesis assumption of a smaller number of joinpoints for each hypothesis test.

## Results

A total of 114 countries had available data for the period 1994–2019. Overall, we estimated 1,064,753 deaths due to Parkinson's disease globally throughout the study period. Far more deaths occurred in men (585,321 deaths) than in women (479,432 deaths), giving a female-to-male ratio of 0.82. Women present 18.09% fewer deaths than men. Between 1994 and 2019, a significant increase in mortality rates was found globally in both men and women. More specifically, the mortality rate (per 100,000 population) in 1994 was 1.76 and reached 5.67 in 2019. The mortality rate in women increased from 1.63 (per 100,000 population) in 1994 to 4.81 in 2019. In men, the mortality rate was 1.91 per 100,000 population in 1994 and 7.88 in 2019.

In data from vital registration systems, huge variations in the patterns of deaths due to Parkinson's disease were observed both over time and between countries. In Japan, for example, the number of deaths due to Parkinson's disease reported in vital registration data increased from 2,427 in 1994 to 10,815 in 2019, so it was noted a 345.61 % increase. Across countries with vital registration data in this period, the proportion of deaths attributed to Parkinson's disease ranged from nearly 0 (Azerbaijan) to 13.52 per 100,000 population (Greece; Supplementary Figure 1).

No records of mortality rates were found between the period of 2008 and 2012 globally. For this reason, we proceeded with the analysis for the periods 1994–2007 and 2013–2019, separately and cumulatively. European countries were mostly among the countries with the highest mortality rates such as Austria, Greece, Germany, Finland, and the United Kingdom with a death rate (per 100,000) of 10.75, 13.52, 9.52, 9.41, and 8.42, respectively, and the United States of America with a mortality rate of 7.69. On the other hand, countries with close to zero mortality rates were also found, such as Egypt (0.05), Jordan (0.05), Iraq (0.06), and Malaysia (0.12; Supplementary Figure 1).

**Figure 1:**
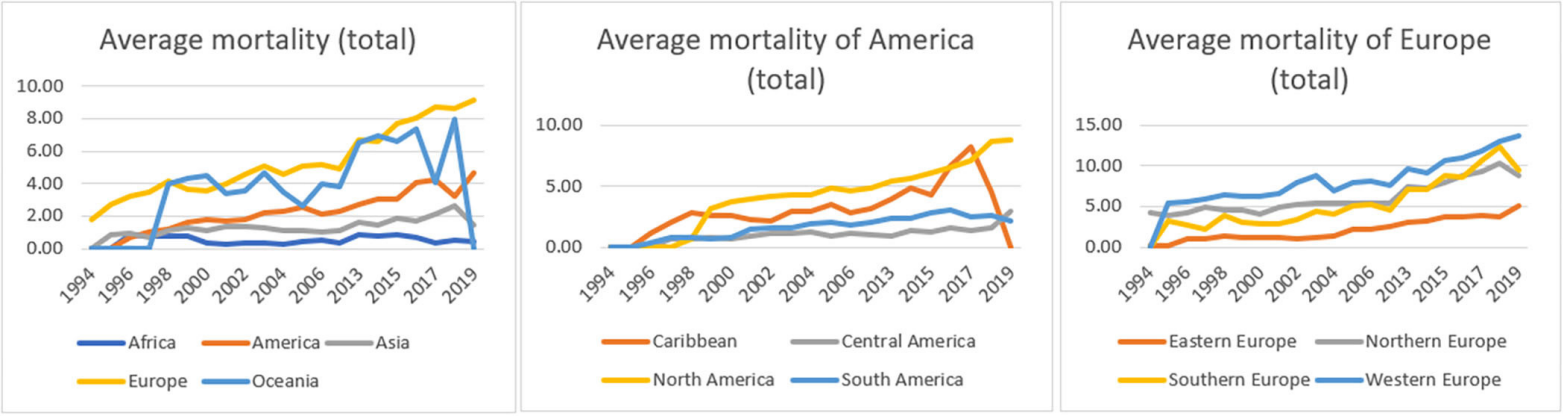
A visual summary of geographical differences of the annual Parkinson's disease-related mortality rate according to age.

**Figure 2:**
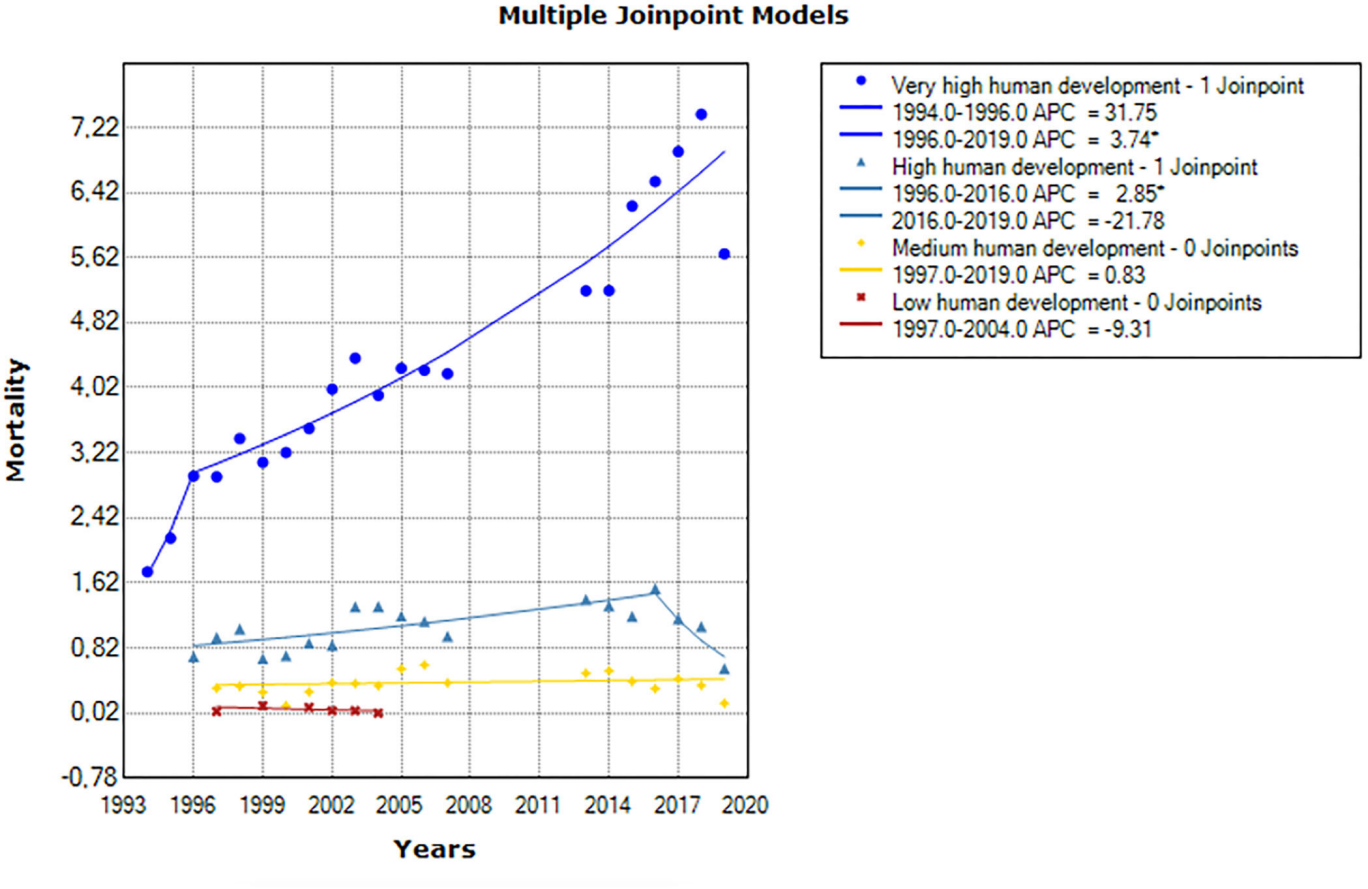
Parkinson's disease-related mortality according to age and according to the classification of the Human Development Index (HDI).

The annual Parkinson's disease-related mortality rate according to age is shown in Table 1. A visual summary of geographical differences is provided in Figure 1. Parkinson's disease-related mortality increased with age, with a seemingly exponential distribution (Figure 2). Total Parkinson's disease deaths, classified by sex for each individual country, are shown in the appendix (Supplementary Figure 2).

**Table 1:** Annual Parkinson's disease-related mortality rate according to age across countries with vital registration data in the period from 1994 to 2019.

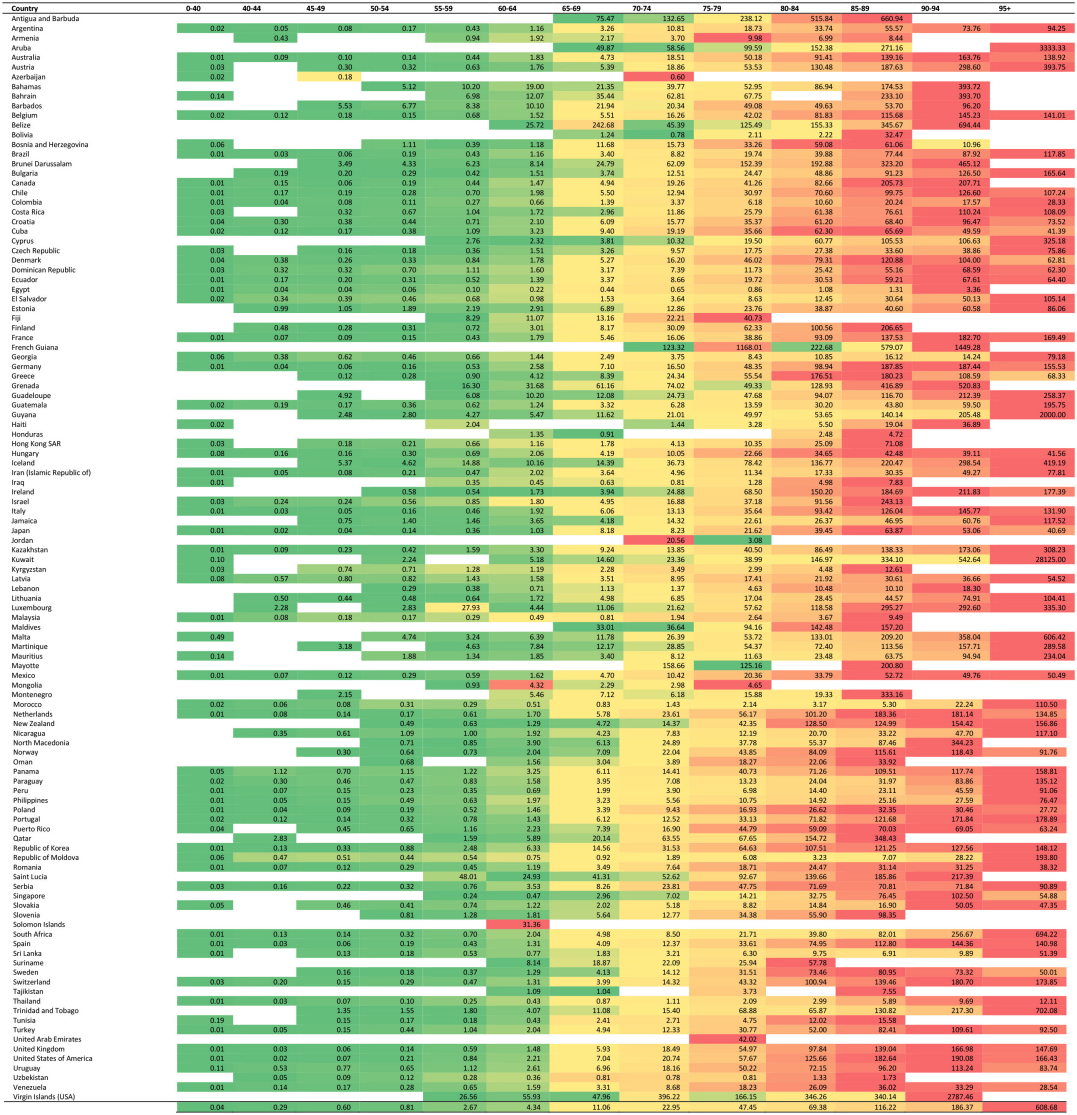

The colours represent the value of the observed annual Parkinson's disease related mortality rates, ranging from green (lowest) to red (highest).

**Figure 3:**
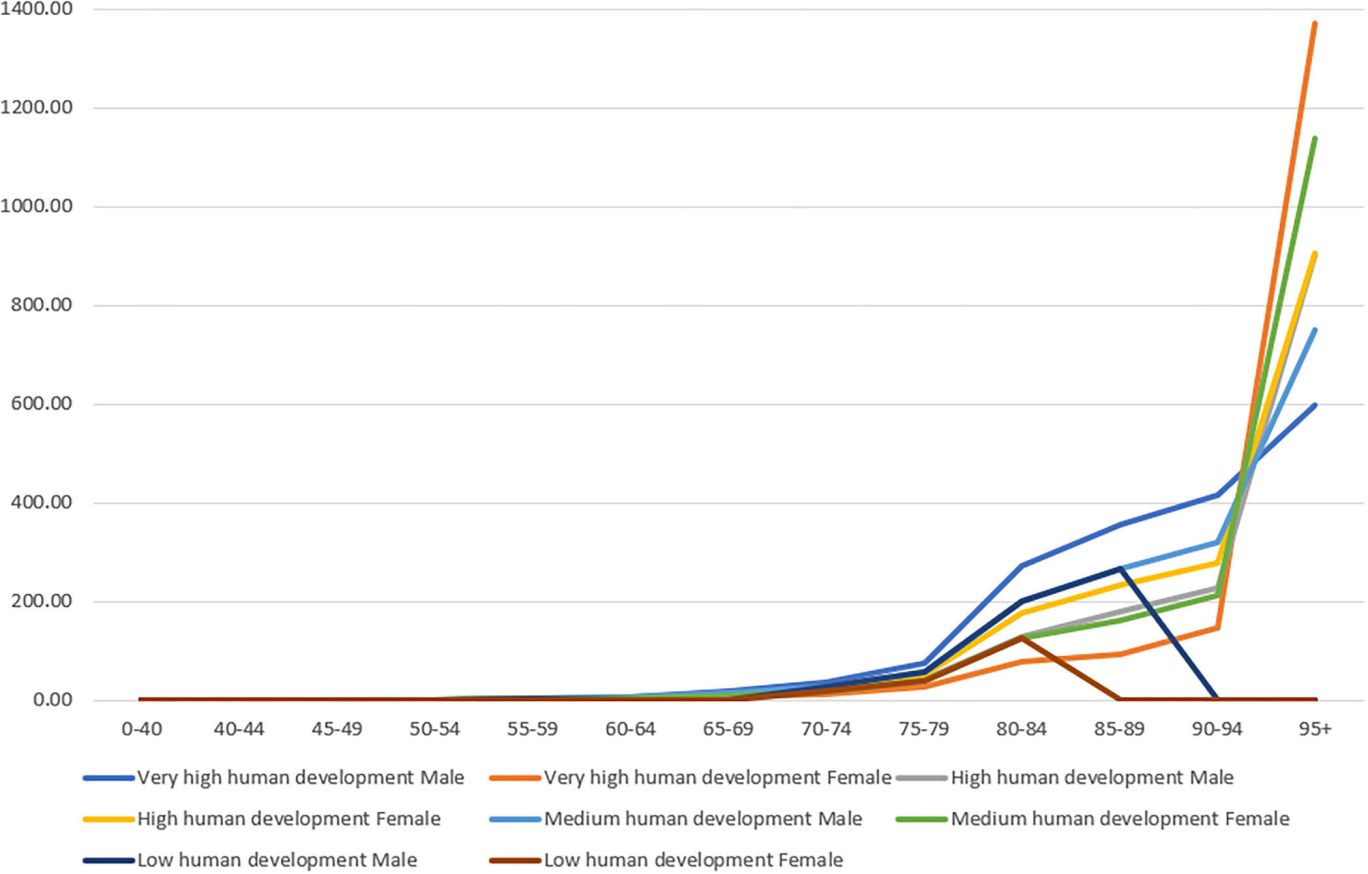
Joinpoint regression analysis showing the differences in the trends of Parkinson's mortality according to the classification of HDI.

Men had higher Parkinson's disease-related mortality rates than women in total and in every age-group separately and consistently for every country. According to the classification of the Human Development Index (HDI), the total average mortality rate is 4.43 per 100,000 population for the very high-human development countries, 1.12 for the high-human development countries, and 0.35 and 0.06 for the medium- and low-human development countries. We performed joinpoint regression analysis in order to assess for differences in the trends of Parkinson's disease mortality according to the classification of the HDI. For countries in the very high-HDI group, there was a non-significant increase by 31.8% (APC = 31.8%, 95%CI:−13.6 to 100.8) between 1994 and 1996 and a significant increase by 3.7% (95% CI: 3.1–4.4) between 1996 and 2019 (Figure 3). Countries in the high-HDI group displayed a significant increase in the rate of Parkinson's disease mortality between 1996 and 2016 (APC = 2.9%, 95% CI: 1.1–4.6). Between 2016 and 2019, we observed a non-significant difference in the annual rate of Parkinson's disease mortality (APC = −21.8; 95% CI: −42.8–7.0). Countries in the medium-HDI group displayed non-significant differences in the trend of the rate of Parkinson's disease mortality throughout the study period (APC = 0.8%, 95% CI:−1.6 – 3.4). Similarly, a non-significant difference was observed for countries in the low-HDI group (APC = −9.3; 95% CI: 33.2–23.2).

We also estimated the average mortality according to geographical distribution on continents. Europe and America displayed a significant increase in the average mortality between 1996 and 2019. More specifically, North America and Western Europe had mostly driven the rising trends as they displayed the most significant differences across the studied period.

Significant differences were found between men and women in every age-group separately and in the total average mortality rate. The total average mortality rate for each group of countries according to the HDI is increasing steadily after the age of 65 years and dramatically after the age of 75 years. This difference was most prominent between countries in the high- and medium-HDI group vs those in the high-HDI group for ages 80–84 years (Figure 2). This difference was very significant for the very high- and medium-development countries and without significant differences for the high-development countries.

Age had a major influence on mortality rate. The highest mortality rate for Parkinson's disease occurred in people aged ≥95 years. Men aged 90–94 years and women aged 75–79 years experienced the fastest annual increase in mortality from Parkinson's disease.

## Discussion

This study provides estimates about the changes in mortality from PD across the world over the past three decades. To our knowledge, no previous studies have focused on the long-term worldwide age- and sex-specific mortality trends in PD. Therefore, our study provides valuable insights into the distinct mortality trends of this neurodegenerative disorder at a population level.

Previous studies on mortality trends of PD are sparse, and their findings ranged widely and have been inconsistent (6, 7, 11). However, most of these studies showed increased mortality in PD. Several observational studies reporting quantitative measures of mortality and postmortem series reporting disease duration at death have been conducted so far (7). A meta-analysis of these studies conducted in 2014 showed that most mortality ratios lay between 1.2 and 2.4 (12).

The global burden of disease study provided a comprehensive overview of PD burden and its trends in the incidence, prevalence, and YLDs at the global, regional, and national levels during 1990–2016. Pronounced increasing trends of PD burden were observed worldwide and in most regions and countries, indicating that PD is an increasing challenge to global health (13). However, no detailed data related to PD mortality are present.

**Figure 4:**
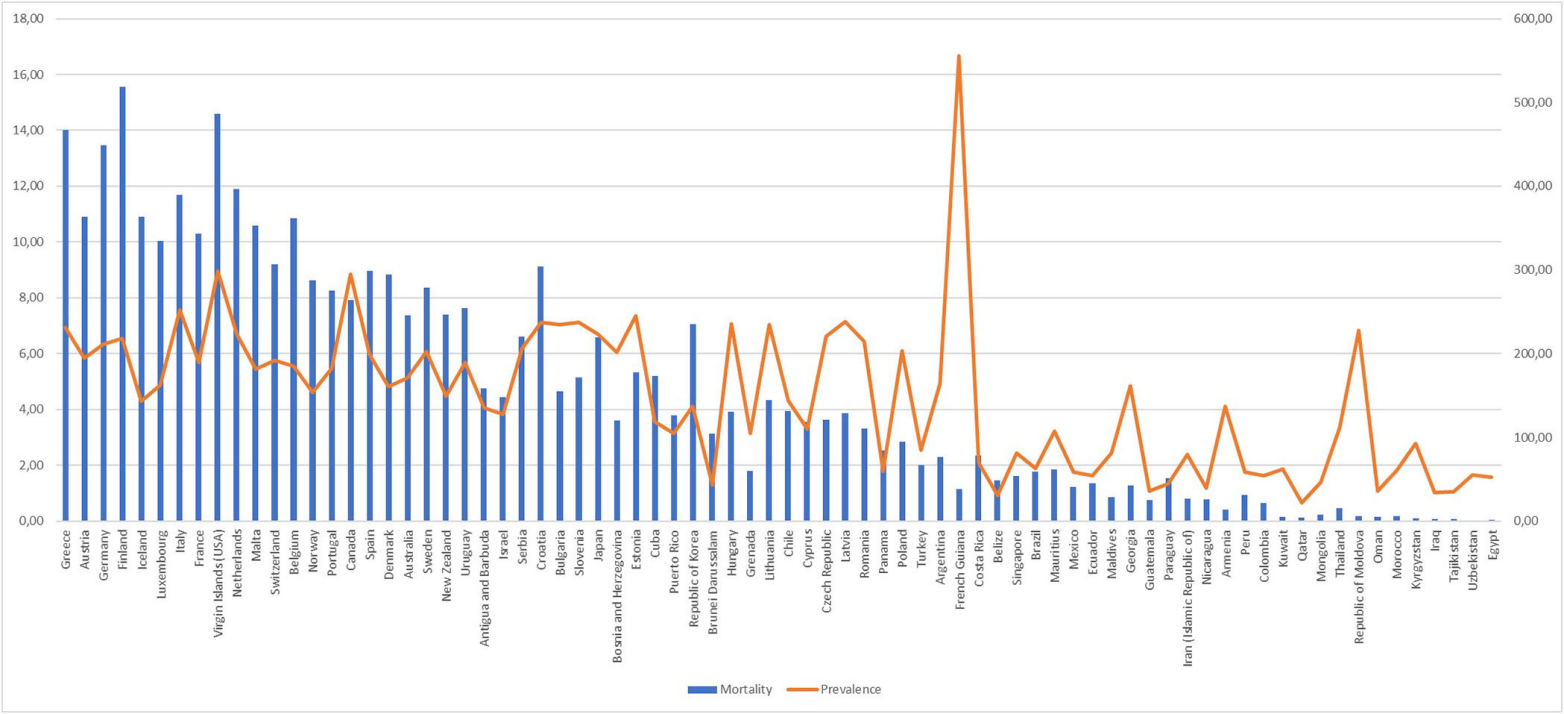
Global prevalence of Parkinson's disease in 2016 in comparison to mortality. Prevalence is expressed as the percentage of the population that is affected by the disease. Data for prevalence were extracted from the “Global, regional, and national burden of Parkinson's disease, 1990–2016: a systematic analysis for the Global Burden of Disease Study 2016”.

Now, more than six million people worldwide have Parkinson's disease, more than doubling in the past generation (3). Parkinson's disease was the neurological condition with the greatest growth rate among those examined by (4). Most of that expansion was caused by aging populations as crude prevalence rates increased by nearly 74% since 1990. Although additional factors, as listed in Figure 4, are likely essential, the increase in prevalence may have a significant impact on mortality. However, the correlation between prevalence and mortality is not consistent across countries.

In the current study, death rates of Parkinson's disease varied by age, sex, and geographic location. Overall, death rates of Parkinson's disease increased significantly from 1994 to 2019. Some interpretations of the results may promote the better understanding of the stable increase in PD mortality. It is possible that increasing incidence in PD over the years led directly to the increase in PD mortality. Increased life expectancy and population aging may also increase the possibility of people developing and being diagnosed with PD. These factors can only partially explain the increase in PD mortality.

The higher socioeconomic status (education level and income) of several countries, such as Europe, could explain the surprisingly increasing mortality rate compared with other countries. Patients in HDI countries are more likely to see an outpatient neurologist; there are more available neurologists specializing in movement disorders; and subsequently, they have a higher chance to receive a diagnosis of PD.

Furthermore, a more precise and early diagnosis due to a better healthcare system in these countries could be a major factor that affects mortality rates. In addition to this, people in countries with very high development according to the HDI have a lower age-adjusted prevalence of major chronic diseases (hypertension, diabetes, cardiovascular disease, stroke, and others) and instead are more likely to develop age-related neurodegenerative diseases. Similar to our findings on PD mortality, a study using the NVSS data has reported higher mortality from Alzheimer's disease, and another in neurodegenerative disease, in White than in other racial/ethnic groups.

Furthermore, recent studies suggested that the improved accuracy of registration practices might affect the results of death certificates and lead to a steady increase in PD mortality (14). This could also partially explain the significant differences between countries according to the HDI. Subsequently, we should emphasize on the fact that quality of data submitted might not be uniform between countries, and this sets a major bias impact. Improvements in the awareness of mortality associated with several neurodegenerative disorders, such as Parkinson's disease and dementia, and clearer guidelines for death certification may have helped the improvement of the quality of data from vital registration systems (15).

Increasing age and male sex appear to be associated with an increased risk of PD previously. Both the incidence and prevalence of PD are 1.5 to 2.0 times higher in men than in women. In the current study, mortality from PD was significantly higher in men than in women during the past three decades for many countries. One possible explanation for this sex difference is that endogenous estrogens, which lead to higher dopamine levels in the striatum, protect women from developing PD (16). Men have been shown to have an earlier onset of PD than women; mortality from the disease occurs earlier, which could also offset other competing risk events in men.

Our study also very clearly showed that the mortality rate dramatically increases with age. Previous studies have shown that mortality rates for geriatric syndromes such as falls and delirium also increase with age (17). Furthermore, older patients with Parkinson's disease often have multiple concomitant conditions, including musculoskeletal disease, cerebrovascular disease, atherosclerosis, heart failure, atrial fibrillation, diabetes, and chronic lung diseases, that complicate the management and may adversely affect outcomes. The presence of multiple disease states can also result in polypharmacy, which is an independent predictor of mortality in the older people (18). Additional important comorbidities in patients with Parkinson's disease include frailty, limited mobility, and falls, which are increasingly recognized as major predictors of mortality in older people (19).

To the best of our knowledge, this is the first population-based study that simultaneously examined the age- and sex-specific mortality trends in Parkinson's disease globally. The strengths of the study are the inclusion of the global population. Furthermore, mortality data collected by the WHO are useful for analyzing contemporary population trends due to its standardized methodology and data quality control.

However, several potential limitations of our study should be considered when interpreting our findings. First, the diagnoses of mortality due to Parkinson's disease were taken from the WHO Mortality Database using ICD-10 codes. Biases due to reporting and misclassification of Parkinson's disease diagnoses are likely to result in an underestimation or overestimation of cases. In reality, depending on who signed the death certificate (general physician vs. neurologist or geriatrician), there could be variation in the awareness of the importance of the disease as a cause of death. In addition, the WHO Mortality Database does not record secondary or contributing causes of death and does not include data on comorbidities and clinical management for each patient, which makes it difficult to precisely state which clinical factors influenced the mortality trends. Another important limitation of our study is that China and India, the most populous countries in the world, do not provide mortality data on Parkinson's disease, and this may significantly affect the total deaths due to this disorder as well as the mortality rate. Despite these limitations, our study provides valuable insights into the distinct mortality trends in Parkinson's disease, which has proven to be an important cause of death globally.

Several other studies in specific populations and in community base Parkinson's disease cohorts confirm our findings. More specifically, a cohort in Norway was followed prospectively from 1993. Independent predictors of mortality during the follow-up were higher age at onset, higher chronological age, male sex, higher Unified Parkinson's Disease Rating Scale (UPDRS) motor score, and presence of psychosis and dementia (20). Another long follow-up study assessing survival in an Austrian PD cohort revealed that male gender, older age at onset, gait disorder, and absence of asymmetry were all associated with decreased survival (21). A study in Italy showed an increasing trend of mortality in the period 1998–2002, rising up to a maximum in 2015, with some differences according to sex and geographical areas (22). Furthermore, the Queensland Parkinson's Project (QPP), an ongoing project that has systematically collected PD data in Australia over the last 20 years, highlighted the increasing mortality of PD over the last 20 years (23).

Using a global death registry, we found a continuous increase in the mortality due to PD in the general population worldwide over the past 3 decades. The increase occurred regardless of age, sex, and geographic location. Continued monitoring and further investigation to understand the underlying reasons for the observed increase in mortality from PD are warranted.

The epidemiological transition matrix is, in large part, defined by the demographic transition. Increases in the population prevalence of older people are accompanied by decreases in the prevalence of infectious diseases, and perinatal, maternal, and child causes of mortality. In addition, life expectancy has increased, and the relationship between population health and age has evolved over time. The wealthiest nations typically have older populations, and because chronic disease and disability are more common in older age-groups, these countries also tend to have higher values for chronic disease mortality rates. However, there are differences in the health of the population between countries and regions. To find aging and health benefits and to develop future policies based on effective and inclusive health systems, it is crucial to comprehend these distinctions.

To sum up, the combination of more sensitive diagnostic criteria that have been established quite recently with the awareness and specialization of more physicians in movement disorders and the lower age-adjusted prevalence of major chronic diseases due to better medical services and emphasis on quality of life factors all contribute to higher mortality rates in patients with PD across HDI countries.

In the context of population aging and growth, the importance of Parkinson's disease as a public health concern will rise (24). Our estimates can be used by healthcare and social care authorities involved in end-of-life and palliative care to improve planning for services addressing the needs of people who die of Parkinson's disease (25). Furthermore, these estimates will aid in directing evidence-based resource allocation and health system planning by assisting funders and policymakers in better understanding the global distribution of Parkinson's disease burden and mortality across regions and time, as well as in making fair comparisons between Parkinson's disease and other diseases.

## Data availability statement

The original contributions presented in the study are included in the article/Supplementary material, further inquiries can be directed to the corresponding author.

## Author contributions

FM, GX, and IL: conceptualization. FM and IL: methodology. IL: formal analysis. IL, OS, KG, and GX: data curation. GX: writing—original draft preparation. FM, OS, KG, and GX: writing—review and editing. All authors have read and agreed to the published version of the manuscript.

## Conflict of interest

The authors declare that the research was conducted in the absence of any commercial or financial relationships that could be construed as a potential conflict of interest.

## Publisher's note

All claims expressed in this article are solely those of the authors and do not necessarily represent those of their affiliated organizations, or those of the publisher, the editors and the reviewers. Any product that may be evaluated in this article, or claim that may be made by its manufacturer, is not guaranteed or endorsed by the publisher.
