# Supplementary material for: Worldwide trends in mortality related to Parkinson's disease in the period of 1994–2019: Analysis of vital registration data from the WHO Mortality Database
Source: Front Neurol. 2022 Oct 4;13:956440. doi: 10.3389/fneur.2022.956440 (PMC9576872; doi:10.3389/fneur.2022.956440)
Supplement: Supplementary file 1 [file Table_1.DOCX]

|  |  | | | |  |  |  |  |  |  |  |  |  |  |  |  |  |  |  |  |  |  |  |
| --- | --- | --- | --- | --- | --- | --- | --- | --- | --- | --- | --- | --- | --- | --- | --- | --- | --- | --- | --- | --- | --- | --- | --- |
|  |  |  |  |  |  |  |  |  |  |  |  |  |  |  |  |  |  |  |  |  |  |  |  |
|  | **Country** | **1994** | **1995** | **1996** | **1997** | **1998** | **1999** | **2000** | **2001** | **2002** | **2003** | **2004** | **2005** | **2006** | **2007** | **2013** | **2014** | **2015** | **2016** | **2017** | **2018** | **2019** | **Average** |
|  | Greece |  |  |  |  |  |  |  |  |  |  |  |  |  |  |  | 8,92 | 11,71 | 14,00 | 16,69 | 16,27 |  | 13,52 |
|  | Austria |  |  |  |  |  |  |  |  | 12,11 | 13,93 | 7,53 | 7,70 | 8,65 | 9,35 | 11,22 | 10,21 | 10,55 | 10,90 | 12,55 | 12,55 | 12,50 | 10,75 |
|  | Germany |  |  |  |  | 6,26 | 6,06 | 6,39 | 6,44 | 7,24 | 7,60 | 7,20 | 6,57 | 7,20 | 6,43 | 11,22 | 11,30 | 13,35 | 13,46 | 14,42 | 15,64 | 15,05 | 9,52 |
|  | Finland |  |  | 6,11 | 6,11 | 6,56 | 7,18 | 6,07 | 6,52 | 7,44 | 7,52 | 7,12 | 7,00 | 7,57 | 7,13 | 10,84 | 13,60 | 14,46 | 15,55 | 15,89 | 16,74 |  | 9,41 |
|  | Iceland |  |  | 3,64 | 8,89 | 8,89 | 7,27 | 4,80 | 6,28 | 6,65 | 8,50 | 9,61 | 10,82 | 8,38 | 8,73 | 14,03 | 6,84 | 9,08 | 10,90 | 13,02 | 16,65 | 13,63 | 9,29 |
|  | Luxembourg |  |  |  |  | 7,81 | 7,06 | 7,71 | 8,71 | 8,71 | 8,46 | 6,47 | 11,37 | 9,84 | 7,21 | 8,10 | 7,69 | 11,16 | 10,04 | 10,23 | 11,34 |  | 8,87 |
|  | Italy |  |  |  |  |  |  |  |  |  | 6,31 | 4,96 | 6,51 | 7,01 | 7,43 | 8,38 | 8,75 | 10,71 | 11,69 | 13,22 |  |  | 8,50 |
|  | United Kingdom |  |  |  |  |  |  |  | 7,52 | 7,85 | 8,37 | 7,54 | 7,91 | 7,80 | 8,42 | 8,69 | 9,37 | 9,42 | 9,74 |  |  |  | 8,42 |
|  | France |  |  |  |  |  |  | 7,07 | 7,45 | 7,43 | 8,20 | 6,78 | 8,25 | 8,17 | 8,30 | 8,73 | 8,93 | 9,60 | 10,29 |  |  |  | 8,27 |
|  | Virgin Islands (USA) |  |  |  |  |  | 9,79 | 1,97 | 1,97 |  | 3,95 | 3,95 | 6,05 |  |  |  |  |  | 14,59 | 20,85 |  |  | 7,89 |
|  | Netherlands |  |  | 5,58 | 6,01 | 5,91 | 6,44 | 4,83 | 5,34 | 6,41 | 7,05 | 6,48 | 6,87 | 7,24 | 6,82 | 8,72 | 8,64 | 10,43 | 11,90 | 12,62 | 12,45 |  | 7,76 |
|  | United States of America |  |  |  |  |  | 5,67 | 5,93 | 6,26 | 6,41 | 6,80 | 6,81 | 6,87 | 6,87 | 7,05 | 8,49 | 8,82 | 9,90 | 10,51 | 11,32 |  |  | 7,69 |
|  | Malta |  | 4,27 | 3,12 | 3,13 | 7,97 | 3,42 | 4,57 | 4,31 | 4,57 | 8,61 | 7,80 | 11,12 | 13,34 | 7,41 | 11,59 | 10,30 | 12,11 | 10,59 | 9,33 |  |  | 7,64 |
|  | Switzerland |  | 5,47 | 5,45 | 5,96 | 5,86 | 5,33 | 5,19 | 5,82 | 8,29 | 8,62 | 7,54 | 7,92 | 8,58 | 8,53 | 9,30 | 8,39 | 10,14 | 9,19 | 9,69 |  |  | 7,52 |
|  | Belgium |  |  |  |  | 6,66 | 6,20 | 5,88 | 5,39 | 6,04 | 7,59 | 6,45 | 6,86 | 7,60 | 6,79 | 9,84 | 8,84 | 9,41 | 10,84 |  |  |  | 7,46 |
|  | Norway |  |  | 6,66 | 8,17 | 7,56 | 7,73 | 7,21 | 7,36 | 6,37 | 6,50 | 7,56 | 5,43 | 5,36 | 5,78 | 7,68 | 9,09 | 8,38 | 8,63 |  |  |  | 7,22 |
|  | Ireland |  |  |  |  |  |  |  |  |  |  |  |  |  | 4,53 | 7,44 | 7,75 | 8,33 |  |  |  |  | 7,01 |
|  | Portugal |  |  |  |  |  |  |  |  | 3,84 | 4,38 |  |  |  | 4,28 | 7,17 | 7,33 | 7,95 | 8,26 | 9,15 | 10,13 |  | 6,94 |
|  | Canada |  |  |  |  |  |  | 4,69 | 5,10 | 5,26 | 5,30 | 5,23 | 6,64 | 5,92 | 6,65 | 6,65 | 6,92 | 7,15 | 7,92 | 8,83 | 8,70 | 8,82 | 6,65 |
|  | Spain |  |  |  |  |  | 4,60 | 4,32 | 4,77 | 4,79 | 5,70 | 5,26 | 6,11 | 6,12 | 6,29 | 7,38 | 7,97 | 8,72 | 8,96 | 9,77 |  |  | 6,48 |
|  | Martinique |  |  |  |  |  |  | 6,20 | 5,39 | 4,04 | 6,47 | 5,12 | 4,53 | 4,28 | 5,04 | 7,23 | 9,30 | 8,86 | 10,24 |  |  |  | 6,39 |
|  | Denmark | 4,28 | 3,87 | 3,05 | 4,46 | 4,64 | 5,88 | 6,28 | 6,22 | 6,50 | 5,97 | 5,05 | 5,16 | 5,71 | 5,69 | 7,06 | 7,39 | 7,76 | 8,82 | 8,08 | 8,77 |  | 6,03 |
|  | Guadeloupe |  |  |  |  |  |  | 3,62 | 3,62 | 4,11 | 5,32 | 3,14 | 4,56 | 5,63 | 6,70 | 7,15 | 10,59 | 7,18 | 9,48 |  |  |  | 5,93 |
|  | Australia |  |  |  |  | 4,02 | 4,33 | 4,43 | 4,61 | 5,12 | 4,42 | 4,90 |  | 5,47 | 6,10 | 7,20 | 7,76 | 7,16 | 7,37 | 7,65 | 7,93 |  | 5,90 |
|  | Sweden |  |  |  | 4,17 | 3,82 | 4,19 | 4,13 | 4,12 | 5,05 | 5,03 | 5,37 | 5,77 | 5,64 | 5,91 | 6,70 | 6,95 | 6,65 | 8,37 | 7,78 | 8,87 |  | 5,79 |
|  | New Zealand |  |  |  |  |  |  | 4,49 | 5,22 | 5,22 | 4,88 | 5,19 | 4,90 | 5,78 | 5,14 | 5,78 | 6,09 | 6,04 | 7,40 |  |  |  | 5,51 |
|  | Uruguay |  |  |  | 2,97 | 2,97 | 2,81 | 3,32 | 4,17 | 4,67 | 4,39 | 4,86 | 6,50 | 5,15 | 6,56 | 7,01 | 7,54 | 7,37 | 7,64 | 7,46 |  |  | 5,34 |
|  | Antigua and Barbuda |  |  |  |  | 6,63 |  | 2,70 |  |  | 9,12 | 6,08 | 5,58 | 2,79 | 2,79 |  | 2,33 |  | 4,75 |  |  |  | 4,75 |
|  | Israel |  |  |  |  | 3,87 | 5,65 | 5,08 | 5,47 | 5,38 | 4,74 | 4,50 | 4,73 | 4,05 | 4,40 | 3,70 | 4,20 | 3,98 | 4,45 | 4,20 | 4,83 |  | 4,58 |
|  | Serbia |  |  |  |  | 2,56 | 3,08 | 3,14 | 2,98 | 3,55 | 3,80 | 3,90 | 4,33 | 4,59 | 4,88 | 5,62 | 4,90 | 5,86 | 6,61 | 7,20 |  |  | 4,47 |
|  | Croatia |  | 2,10 | 2,15 | 1,58 | 1,92 | 1,85 | 2,32 | 1,97 | 2,22 | 2,22 | 2,90 | 4,53 | 5,52 | 5,40 | 6,51 | 6,77 | 8,96 | 9,13 | 11,24 |  |  | 4,41 |
|  | Bulgaria |  |  |  |  |  |  |  |  |  |  |  | 4,04 | 4,01 | 5,04 | 4,38 | 5,24 | 4,41 | 4,66 | 3,66 | 3,34 |  | 4,31 |
|  | Slovenia |  |  |  | 2,03 | 2,88 | 2,67 | 2,34 | 2,56 | 3,76 | 3,21 | 2,67 | 2,26 | 2,36 | 2,51 | 4,74 | 4,96 | 4,06 | 5,15 | 8,76 | 10,59 | 9,45 | 4,27 |
|  | Japan |  | 1,94 | 1,83 | 1,99 | 1,98 | 2,11 | 2,36 | 2,49 | 2,55 | 2,66 | 2,75 | 3,03 | 3,21 | 3,40 | 5,36 | 5,36 | 6,25 | 6,58 | 8,83 | 9,43 |  | 3,90 |
|  | Bosnia and Herzegovina |  |  |  |  |  |  |  |  |  |  |  |  |  |  |  | 3,99 |  | 3,62 |  |  |  | 3,80 |
|  | Aruba |  |  |  |  |  | 2,87 |  |  |  | 2,28 |  | 2,20 | 3,31 | 3,81 | 3,93 | 7,87 | 2,88 | 3,83 |  |  |  | 3,67 |
|  | Estonia |  |  |  | 1,57 | 1,90 | 1,65 | 1,68 | 2,61 | 1,94 | 1,77 | 2,53 | 2,21 | 2,67 | 2,98 | 4,58 | 4,65 | 5,73 | 5,33 | 6,77 | 8,12 | 6,37 | 3,61 |
|  | Cuba |  |  |  |  |  |  |  | 1,99 | 1,98 | 2,17 | 3,04 | 3,28 | 2,66 | 2,98 | 4,20 | 4,78 | 5,15 | 5,21 | 5,83 |  |  | 3,60 |
|  | Puerto Rico |  |  |  |  |  | 3,01 | 2,97 | 3,35 | 3,54 | 3,42 | 2,91 | 3,60 | 2,75 | 3,66 | 4,55 | 4,69 | 3,72 | 3,79 | 4,48 |  |  | 3,60 |
|  | Barbados |  |  |  |  |  |  | 4,72 | 1,97 | 2,76 | 1,57 | 2,36 | 6,20 | 4,65 | 3,25 | 4,17 |  |  |  |  |  |  | 3,52 |
|  | Republic of Korea |  | 0,39 | 0,49 | 0,62 | 0,68 | 0,84 | 1,18 | 1,53 | 2,06 | 1,95 | 2,28 | 2,44 | 2,63 | 2,74 | 6,37 | 6,71 | 6,69 | 7,06 | 7,31 | 7,77 | 6,84 | 3,43 |
|  | North Macedonia |  |  |  |  |  |  |  |  |  |  |  |  | 2,51 | 1,72 | 5,44 |  |  |  |  |  |  | 3,22 |
|  | Brunei Darussalam |  |  |  |  |  |  |  |  |  |  |  |  |  |  | 3,42 | 2,28 | 3,37 | 3,13 | 3,37 | 1,21 | 4,58 | 3,05 |
|  | Belarus |  |  |  |  |  |  |  |  |  |  |  |  |  |  |  |  |  |  |  | 2,93 |  | 2,93 |
|  | Hungary |  |  | 1,76 | 1,79 | 2,29 | 1,96 | 2,22 | 2,16 | 1,49 | 1,71 | 1,79 | 2,20 | 2,11 | 2,59 | 3,40 | 3,58 | 4,47 | 3,92 | 4,95 | 4,98 | 5,07 | 2,87 |
|  | Grenada |  |  |  |  |  |  |  |  | 1,95 | 3,93 | 1,97 | 1,92 | 4,04 | 2,13 | 3,81 | 2,08 | 2,74 | 1,81 | 1,84 | 4,56 |  | 2,73 |
|  | Lithuania |  |  |  |  | 0,36 | 0,36 | 0,47 | 0,61 | 1,11 | 1,11 | 1,43 | 1,94 | 2,36 | 2,75 | 3,30 | 3,68 | 3,92 | 4,34 | 5,73 | 6,07 | 6,39 | 2,70 |
|  | Saint Lucia |  |  | 2,05 | 5,60 | 1,37 | 2,05 |  | 1,91 | 1,25 | 1,30 | 5,10 | 2,49 | 1,22 |  | 4,32 | 3,17 | 3,10 |  |  |  |  | 2,69 |
|  | Chile |  |  |  | 0,83 | 0,57 | 0,39 | 1,01 | 1,94 | 2,11 | 2,19 | 2,76 | 2,73 | 2,69 | 3,20 | 4,79 | 4,13 | 3,95 | 3,96 | 4,37 | 3,77 |  | 2,67 |
|  | Cyprus |  |  |  |  |  |  |  |  |  |  | 2,36 | 2,47 | 1,95 | 1,95 | 2,48 | 2,39 | 2,75 | 3,54 | 2,39 | 3,72 |  | 2,60 |
|  | Kazakhstan |  |  |  |  |  |  |  |  |  |  |  |  |  |  | 1,69 | 1,98 | 3,01 |  | 3,20 |  |  | 2,47 |
|  | Trinidad and Tobago |  |  |  |  |  | 2,09 | 1,96 | 3,59 | 2,29 | 2,29 | 1,96 | 3,39 | 2,55 | 1,93 |  |  |  |  |  |  |  | 2,45 |
|  | Czech Republic | 0,84 | 0,56 | 0,59 | 0,67 | 0,98 | 1,17 | 1,66 | 2,05 | 2,05 | 2,45 | 2,30 | 2,76 | 2,37 | 1,12 | 2,88 | 2,72 | 3,19 | 3,64 | 3,90 | 4,08 | 4,37 | 2,21 |
|  | Latvia |  |  | 0,62 | 0,41 | 1,23 | 0,45 | 1,52 | 2,92 | 2,96 | 2,48 | 1,31 | 1,12 | 1,41 | 1,07 | 2,45 | 2,18 | 2,73 | 3,87 | 5,21 | 5,11 |  | 2,17 |
|  | Romania |  |  |  |  |  | 0,82 | 1,09 | 0,91 | 0,98 | 1,32 | 1,34 | 1,68 | 1,73 | 1,64 | 2,72 | 2,87 | 3,23 | 3,32 | 3,51 | 3,71 |  | 2,06 |
|  | Panama |  |  |  |  | 1,37 | 1,41 | 1,16 | 1,56 | 2,24 | 2,34 | 2,17 | 1,98 | 1,70 | 2,24 | 1,95 | 2,92 | 2,52 | 2,52 | 2,39 | 2,39 |  | 2,05 |
|  | Poland |  |  |  |  |  | 0,87 | 0,96 | 0,94 | 0,99 | 1,06 | 1,22 | 2,03 | 1,91 | 2,13 | 2,80 | 2,45 | 2,69 | 2,83 | 3,35 | 3,39 |  | 1,97 |
|  | Mayotte |  |  |  |  |  |  |  |  |  |  |  |  |  |  | 3,02 | 1,01 | 2,50 | 0,83 |  |  |  | 1,84 |
|  | Turkey |  |  |  |  |  |  |  |  |  |  |  |  |  |  | 1,57 | 1,83 | 1,87 | 2,00 |  |  | 1,75 | 1,81 |
|  | Argentina |  |  |  | 1,56 | 1,59 | 1,67 | 1,68 | 1,50 | 1,55 | 1,84 | 1,65 | 1,55 | 1,56 | 1,98 | 2,08 | 1,86 | 1,95 | 2,30 | 2,18 | 2,03 |  | 1,80 |
|  | French Guiana |  |  |  |  |  |  |  | 1,32 | 2,64 | 2,07 | 2,64 | 1,99 | 0,99 | 1,18 | 2,00 | 1,00 | 0,77 | 1,16 |  |  |  | 1,62 |
|  | Bahamas |  |  |  |  |  | 0,78 | 0,72 | 0,68 | 1,03 | 1,37 | 3,08 | 3,47 | 0,69 | 1,61 | 1,75 | 2,33 | 1,34 |  |  |  |  | 1,57 |
|  | Jamaica |  |  |  |  |  |  | 1,45 | 1,76 | 1,96 | 1,41 | 1,49 | 1,13 | 1,21 |  | 1,72 | 1,95 |  |  |  |  |  | 1,57 |
|  | Costa Rica |  |  |  | 0,98 | 0,95 | 1,35 | 0,86 | 1,06 | 1,14 | 1,20 | 1,77 | 1,49 | 1,76 | 1,34 | 1,27 | 1,60 | 1,94 | 2,35 | 1,90 | 1,88 | 3,00 | 1,55 |
|  | Hong Kong SAR |  |  |  |  |  |  |  | 1,07 | 1,11 | 1,48 | 1,30 | 1,32 | 1,31 | 1,60 | 1,69 | 1,69 | 1,58 | 2,07 | 1,57 |  |  | 1,48 |
|  | Belize |  |  |  | 1,10 |  | 0,97 | 1,31 | 1,31 | 1,75 | 1,31 | 1,89 | 1,08 | 2,16 | 1,44 | 1,09 | 1,45 | 1,23 | 1,46 |  |  |  | 1,40 |
|  | Singapore |  |  |  |  |  |  |  |  |  |  |  |  |  |  | 1,12 | 0,86 | 1,36 | 1,61 | 1,44 |  | 1,48 | 1,31 |
|  | Brazil |  |  | 0,42 | 0,52 | 0,54 | 0,59 | 0,63 | 0,73 | 0,77 | 0,87 | 0,96 | 1,02 | 1,18 | 1,19 | 1,71 | 1,89 | 1,64 | 1,76 | 1,84 | 2,05 | 2,21 | 1,19 |
|  | Mauritius |  |  |  |  |  |  |  |  |  |  |  | 0,49 | 0,98 | 0,74 | 0,99 | 1,91 | 0,70 | 1,85 | 0,35 | 1,05 | 0,88 | 0,99 |
|  | Mexico |  |  |  |  | 0,74 | 0,74 | 0,71 | 0,68 | 0,80 | 0,88 | 0,88 | 1,06 | 1,07 | 0,98 | 1,04 | 1,12 | 1,21 | 1,22 | 1,25 |  |  | 0,96 |
|  | Guyana |  |  |  |  |  |  |  | 0,56 | 1,07 | 0,98 | 0,84 | 1,30 | 1,59 | 0,72 | 0,97 | 0,55 |  |  |  |  |  | 0,95 |
|  | Ecuador |  |  |  | 0,53 | 0,66 | 0,67 | 0,84 | 0,64 | 0,44 | 0,50 | 0,76 | 0,90 | 0,85 | 0,98 | 1,26 | 1,14 | 1,17 | 1,36 | 1,42 |  |  | 0,88 |
|  | Maldives |  |  |  |  |  |  |  |  | 1,75 |  |  |  |  |  | 0,60 |  | 0,43 | 0,87 | 0,72 |  |  | 0,88 |
|  | Slovakia | 0,16 | 0,14 | 0,14 | 0,30 | 0,46 | 0,78 | 0,79 | 0,69 | 0,91 | 0,95 | 0,93 | 1,13 | 0,93 | 1,28 | 1,62 | 2,16 |  |  |  |  |  | 0,84 |
|  | Georgia |  |  |  |  | 0,36 | 0,22 | 0,21 | 0,19 |  |  | 0,12 | 0,20 | 0,18 | 0,08 | 1,37 | 1,34 | 1,62 | 1,29 | 1,64 | 1,40 | 1,73 | 0,79 |
|  | Montenegro |  |  |  |  |  |  | 0,84 | 1,01 | 0,67 | 0,66 | 1,01 | 0,96 | 0,34 | 0,66 |  |  |  |  |  |  |  | 0,77 |
|  | South Africa |  |  | 0,88 | 0,73 | 0,78 | 0,79 | 0,79 | 0,74 | 0,78 | 0,81 | 0,68 | 0,64 | 0,64 |  | 0,57 | 0,63 | 0,78 |  |  |  |  | 0,73 |
|  | Venezuela |  |  | 0,47 | 0,62 | 0,49 | 0,66 | 0,57 | 0,59 | 0,69 | 0,88 | 0,59 | 0,78 | 0,54 | 0,73 | 0,90 | 0,98 |  |  |  |  |  | 0,68 |
|  | Guatemala |  |  |  |  |  |  |  |  |  |  |  | 0,55 | 0,43 | 0,61 | 0,66 | 0,59 | 0,81 | 0,75 | 0,64 |  |  | 0,63 |
|  | Dominican Republic |  |  | 0,23 | 0,62 | 0,41 | 0,51 | 0,47 | 0,61 | 0,56 | 0,63 | 0,89 | 0,78 | 0,84 | 0,77 | 0,45 |  |  |  |  |  |  | 0,60 |
|  | Suriname |  |  |  | 0,47 | 0,70 | 0,47 | 0,43 | 0,86 | 0,42 | 0,43 |  | 1,21 | 0,40 | 0,40 | 0,81 | 0,40 |  |  |  |  |  | 0,58 |
|  | Bahrain |  |  |  | 0,31 | 0,62 | 0,31 |  |  | 0,69 |  | 0,35 | 0,72 | 0,42 | 1,04 | 0,78 | 0,29 |  |  |  |  |  | 0,55 |
|  | Paraguay |  |  | 0,23 | 0,10 | 0,15 | 0,10 | 0,19 | 0,15 | 0,13 | 0,11 | 0,24 | 0,28 | 0,37 | 0,53 | 1,15 | 1,22 | 1,44 | 1,54 | 1,31 |  |  | 0,54 |
|  | Solomon Islands |  |  |  |  |  |  |  |  |  |  |  |  |  |  |  |  |  |  | 0,54 |  |  | 0,54 |
|  | Iran (Islamic Republic of) |  |  |  |  |  |  |  |  |  |  |  |  |  |  | 0,21 | 0,36 | 0,77 | 0,82 |  |  |  | 0,54 |
|  | Tunisia |  |  |  |  |  |  |  |  |  |  |  |  |  |  | 0,40 |  |  |  | 0,65 |  |  | 0,52 |
|  | Nicaragua |  |  |  | 0,44 | 0,51 | 0,38 | 0,11 | 0,23 | 0,23 | 0,45 | 0,36 | 0,40 | 0,40 | 0,40 | 0,87 | 0,83 | 0,48 | 0,78 | 0,55 | 0,64 |  | 0,47 |
|  | Armenia |  |  |  |  |  |  |  |  |  |  |  |  | 0,54 | 0,68 | 0,37 | 0,55 | 0,44 | 0,40 | 0,63 | 0,22 | 0,15 | 0,44 |
|  | El Salvador |  |  |  | 0,21 | 0,19 | 0,17 | 0,16 | 0,42 | 0,37 | 0,30 | 0,33 | 0,51 | 0,43 | 0,38 | 0,72 | 0,85 | 0,90 |  |  |  |  | 0,42 |
|  | Peru |  |  |  |  |  | 0,15 | 0,43 | 0,30 | 0,32 | 0,47 | 0,46 | 0,21 | 0,44 | 0,61 | 0,08 | 0,05 | 0,03 | 0,94 | 0,95 |  |  | 0,39 |
|  | Colombia |  |  |  | 0,18 | 0,20 | 0,28 | 0,21 | 0,19 | 0,28 | 0,24 | 0,32 | 0,33 | 0,25 | 0,33 | 0,42 | 0,48 | 0,63 | 0,65 | 0,53 |  |  | 0,35 |
|  | Sri Lanka |  |  |  |  |  |  |  |  |  |  | 0,25 | 0,18 | 0,34 | 0,28 | 0,57 | 0,44 |  |  |  |  |  | 0,34 |
|  | Kuwait |  | 0,58 | 0,39 | 0,45 | 0,45 | 0,57 | 0,65 | 0,50 | 0,38 | 0,38 | 0,49 | 0,15 | 0,20 | 0,25 | 0,07 | 0,08 | 0,08 | 0,15 | 0,22 |  |  | 0,33 |
|  | Philippines |  |  |  |  |  | 0,28 | 0,28 | 0,32 | 0,29 | 0,36 |  |  | 0,32 | 0,35 |  | 0,45 |  |  |  |  |  | 0,33 |
|  | Fiji |  |  |  |  |  |  |  | 0,25 | 0,26 |  | 0,26 | 0,29 | 0,58 | 0,29 |  |  |  |  |  |  |  | 0,32 |
|  | Qatar |  | 0,59 |  |  |  |  |  |  |  |  |  | 0,21 | 0,26 | 0,53 | 0,12 | 0,23 |  | 0,12 |  |  |  | 0,29 |
|  | Mongolia |  |  |  |  |  |  |  |  |  |  |  |  |  |  |  |  |  | 0,24 |  |  |  | 0,24 |
|  | Lebanon |  |  |  |  |  |  |  |  |  |  |  |  |  |  |  |  |  |  | 0,24 | 0,21 | 0,26 | 0,24 |
|  | Thailand |  |  |  | 0,01 | 0,01 | 0,01 | 0,01 |  | 0,03 | 0,04 | 0,02 | 0,04 | 0,04 | 0,05 | 0,24 | 0,30 | 0,35 | 0,47 | 0,39 | 0,44 | 0,68 | 0,19 |
|  | Republic of Moldova |  |  |  | 0,14 | 0,24 | 0,05 | 0,09 | 0,17 | 0,10 | 0,25 | 0,05 | 0,12 | 0,15 | 0,10 | 0,20 | 0,20 | 0,05 | 0,19 | 0,15 | 0,15 |  | 0,14 |
|  | Oman |  |  |  |  |  |  |  |  |  |  |  |  |  |  |  | 0,20 |  | 0,14 | 0,07 |  | 0,12 | 0,13 |
|  | Morocco |  |  |  |  |  |  | 0,09 | 0,02 | 0,10 | 0,11 | 0,10 |  |  | 0,16 | 0,21 | 0,21 | 0,17 | 0,18 |  |  |  | 0,13 |
|  | Malaysia |  |  |  |  |  |  | 0,10 | 0,15 | 0,11 | 0,14 | 0,16 | 0,16 | 0,11 | 0,11 | 0,04 | 0,09 |  |  |  |  |  | 0,12 |
|  | Kyrgyzstan |  |  |  |  |  |  | 0,09 | 0,17 | 0,20 | 0,09 | 0,15 | 0,06 | 0,21 | 0,08 | 0,09 | 0,19 | 0,04 | 0,10 | 0,03 | 0,07 | 0,14 | 0,12 |
|  | United Arab Emirates |  |  |  |  |  |  |  |  |  |  |  |  |  |  |  |  |  |  |  |  | 0,07 | 0,07 |
|  | Iraq |  |  |  |  |  |  |  |  |  |  |  |  |  |  |  |  | 0,06 | 0,07 |  |  |  | 0,06 |
|  | Honduras |  |  |  |  |  |  |  |  |  |  |  |  |  |  | 0,06 |  |  |  |  |  |  | 0,06 |
|  | Tajikistan |  |  |  |  |  |  |  |  |  |  |  |  |  |  |  |  |  | 0,06 |  |  |  | 0,06 |
|  | Haiti |  |  |  | 0,04 |  | 0,11 |  | 0,09 | 0,05 | 0,05 | 0,02 |  |  |  |  |  |  |  |  |  |  | 0,06 |
|  | Uzbekistan |  |  |  |  |  |  |  |  |  |  | 0,04 | 0,03 |  |  |  | 0,05 | 0,10 | 0,02 | 0,04 | 0,07 | 0,06 | 0,05 |
|  | Egypt |  |  |  |  |  |  | 0,04 | 0,04 | 0,06 | 0,07 | 0,05 | 0,06 | 0,06 | 0,05 | 0,03 | 0,04 | 0,06 | 0,04 | 0,05 | 0,04 | 0,04 | 0,05 |
|  | Bolivia |  |  |  |  |  |  | 0,03 | 0,06 | 0,03 | 0,08 |  |  |  |  |  |  |  |  |  |  |  | 0,05 |
|  | Jordan |  |  |  |  |  |  |  |  |  |  |  |  |  |  |  |  | 0,05 |  |  |  |  | 0,05 |
|  | Azerbaijan |  |  |  |  |  |  |  |  |  |  |  |  |  | 0,04 |  |  |  |  |  |  |  | 0,04 |
|  |  | 1,76 | 1,99 | 2,18 | 2,01 | 2,54 | 2,40 | 2,38 | 2,50 | 2,75 | 3,15 | 2,90 | 3,14 | 3,04 | 3,05 | 3,81 | 3,91 | 4,48 | 4,87 | 5,26 | 5,73 | 4,23 |  |

Supplementary Figure 1. The proportion of deaths attributed to Parkinson’s disease per 100.000 across countries with vital registration data in the period from 1994 to 2019

|  | | | | |
| --- | --- | --- | --- | --- |
|  |  |  |  |  |
| Country | Total |  | Man | Woman |
| Antigua and Barbuda | 4,75 |  | 5,39 | 2,52 |
| Argentina | 1,80 |  | 1,96 | 1,64 |
| Armenia | 0,44 |  | 0,47 | 0,42 |
| Aruba | 3,67 |  | 4,43 | 3,22 |
| Australia | 5,90 |  | 7,33 | 4,55 |
| Austria | 10,75 |  | 11,63 | 10,06 |
| Azerbaijan | 0,04 |  | 0,03 | 0,05 |
| Bahamas | 1,57 |  | 1,97 | 1,19 |
| Bahrain | 0,55 |  | 0,55 | 0,59 |
| Barbados | 3,52 |  | 3,88 | 3,18 |
| Belarus | 2,93 |  | 2,91 | 2,95 |
| Belgium | 7,46 |  | 8,08 | 6,90 |
| Belize | 1,40 |  | 1,69 | 1,12 |
| Bolivia | 0,05 |  | 0,06 | 0,04 |
| Bosnia and Herzegovina | 3,80 |  | 4,43 | 3,27 |
| Brazil | 1,19 |  | 1,36 | 1,03 |
| Brunei Darussalam | 3,05 |  | 3,90 | 2,19 |
| Bulgaria | 4,31 |  | 5,03 | 3,66 |
| Canada | 6,65 |  | 8,56 | 5,12 |
| Chile | 2,67 |  | 2,93 | 2,44 |
| Colombia | 0,35 |  | 0,39 | 0,30 |
| Costa Rica | 1,55 |  | 1,86 | 1,27 |
| Croatia | 4,41 |  | 4,80 | 4,07 |
| Cuba | 3,60 |  | 4,88 | 2,48 |
| Cyprus | 2,60 |  | 3,19 | 2,05 |
| Czech Republic | 2,21 |  | 2,82 | 1,68 |
| Denmark | 6,03 |  | 6,90 | 5,22 |
| Dominican Republic | 0,60 |  | 0,79 | 0,44 |
| Ecuador | 0,88 |  | 1,06 | 0,71 |
| Egypt | 0,05 |  | 0,06 | 0,04 |
| El Salvador | 0,42 |  | 0,57 | 0,30 |
| Estonia | 3,61 |  | 3,78 | 3,50 |
| Fiji | 0,32 |  | 0,34 | 0,25 |
| Finland | 9,41 |  | 10,52 | 8,51 |
| France | 8,27 |  | 10,17 | 6,80 |
| French Guiana | 1,62 |  | 1,69 | 1,10 |
| Georgia | 0,79 |  | 0,91 | 0,70 |
| Germany | 9,52 |  | 11,36 | 7,96 |
| Greece | 13,52 |  | 15,61 | 11,81 |
| Grenada | 2,73 |  | 2,61 | 3,06 |
| Guadeloupe | 5,93 |  | 8,32 | 3,95 |
| Guatemala | 0,63 |  | 0,83 | 0,47 |
| Guyana | 0,95 |  | 1,27 | 0,75 |
| Haiti | 0,06 |  | 0,07 | 0,05 |
| Honduras | 0,06 |  | 0,02 | 0,11 |
| Hong Kong SAR | 1,48 |  | 1,75 | 1,27 |
| Hungary | 2,87 |  | 3,64 | 2,28 |
| Iceland | 9,29 |  | 10,80 | 7,91 |
| Iran (Islamic Republic of) | 0,54 |  | 0,75 | 0,35 |
| Iraq | 0,06 |  | 0,07 | 0,06 |
| Ireland | 7,01 |  | 8,52 | 5,65 |
| Israel | 4,58 |  | 5,61 | 3,73 |
| Italy | 8,50 |  | 9,66 | 7,51 |
| Jamaica | 1,57 |  | 1,95 | 1,22 |
| Japan | 3,90 |  | 4,02 | 3,80 |
| Jordan | 0,05 |  | 0,05 |  |
| Kazakhstan | 2,47 |  | 2,12 | 2,77 |
| Kuwait | 0,33 |  | 0,41 | 0,24 |
| Kyrgyzstan | 0,12 |  | 0,11 | 0,13 |
| Latvia | 2,17 |  | 2,13 | 2,21 |
| Lebanon | 0,24 |  | 0,26 | 0,22 |
| Lithuania | 2,70 |  | 2,61 | 2,78 |
| Luxembourg | 8,87 |  | 9,54 | 8,27 |
| Malaysia | 0,12 |  | 0,15 | 0,08 |
| Maldives | 0,88 |  | 0,82 | 0,84 |
| Malta | 7,64 |  | 9,11 | 6,56 |
| Martinique | 6,39 |  | 8,53 | 4,66 |
| Mauritius | 0,99 |  | 1,09 | 0,91 |
| Mayotte | 1,84 |  | 2,07 | 1,23 |
| Mexico | 0,96 |  | 1,16 | 0,78 |
| Mongolia | 0,24 |  | 0,44 | 0,07 |
| Montenegro | 0,77 |  | 0,66 | 0,95 |
| Morocco | 0,13 |  | 0,16 | 0,10 |
| Netherlands | 7,76 |  | 9,17 | 6,66 |
| New Zealand | 5,51 |  | 7,22 | 4,08 |
| Nicaragua | 0,47 |  | 0,66 | 0,32 |
| North Macedonia | 3,22 |  | 4,34 | 2,19 |
| Norway | 7,22 |  | 8,31 | 6,31 |
| Oman | 0,13 |  | 0,15 | 0,11 |
| Panama | 2,05 |  | 2,39 | 1,74 |
| Paraguay | 0,54 |  | 0,59 | 0,49 |
| Peru | 0,39 |  | 0,50 | 0,29 |
| Philippines | 0,33 |  | 0,38 | 0,29 |
| Poland | 1,97 |  | 2,10 | 1,87 |
| Portugal | 6,94 |  | 8,14 | 5,90 |
| Puerto Rico | 3,60 |  | 5,32 | 2,37 |
| Qatar | 0,29 |  | 0,27 | 0,34 |
| Republic of Korea | 3,43 |  | 2,93 | 3,79 |
| Republic of Moldova | 0,14 |  | 0,17 | 0,12 |
| Romania | 2,06 |  | 2,46 | 1,72 |
| Saint Lucia | 2,69 |  | 3,48 | 1,82 |
| Serbia | 4,47 |  | 5,42 | 3,66 |
| Singapore | 1,31 |  | 1,54 | 1,10 |
| Slovakia | 0,84 |  | 1,04 | 0,67 |
| Slovenia | 4,27 |  | 4,92 | 3,72 |
| Solomon Islands | 0,54 |  | 0,54 |  |
| South Africa | 0,73 |  | 0,91 | 0,58 |
| Spain | 6,48 |  | 6,90 | 6,11 |
| Sri Lanka | 0,34 |  | 0,41 | 0,29 |
| Suriname | 0,58 |  | 0,62 | 0,57 |
| Sweden | 5,79 |  | 6,69 | 5,01 |
| Switzerland | 7,52 |  | 8,69 | 6,48 |
| Tajikistan | 0,06 |  | 0,10 | 0,02 |
| Thailand | 0,19 |  | 0,21 | 0,19 |
| Trinidad and Tobago | 2,45 |  | 3,05 | 1,89 |
| Tunisia | 0,52 |  | 0,56 | 0,49 |
| Turkey | 1,81 |  | 2,12 | 1,52 |
| United Arab Emirates | 0,07 |  |  | 0,07 |
| United Kingdom | 8,42 |  | 10,58 | 6,56 |
| United States of America | 7,69 |  | 9,54 | 6,06 |
| Uruguay | 5,34 |  | 5,98 | 4,77 |
| Uzbekistan | 0,05 |  | 0,06 | 0,05 |
| Venezuela | 0,68 |  | 0,79 | 0,57 |
| Virgin Islands (USA) | 7,89 |  | 6,89 |  |

Supplementary Figure 2. Total and Parkinson’s disease deaths, classified by sex for each individual country
